# Impact of deafness on numerical tasks implying visuospatial and verbal processes

**DOI:** 10.1038/s41598-022-14728-3

**Published:** 2022-07-01

**Authors:** Margot Buyle, Valentina Vencato, Virginie Crollen

**Affiliations:** Psychological Science Research Institute (IPSY) and Institute of Neuroscience (IoNS), Université Catholique de Louvain, Place Cardinal Mercier 10, 1348 Louvain-la-Neuve, Belgium

**Keywords:** Neuroscience, Psychology

## Abstract

The literature suggests that deaf individuals lag behind their hearing peers in terms of mathematical abilities. However, it is still unknown how unique sensorimotor experiences, like deafness, might shape number-space interactions. We still do not know either the spatial frame of reference deaf individuals use to map numbers onto space in different numerical tasks. To examine these issues, deaf, hearing signer and hearing control adults were asked to perform a number comparison and a parity judgment task with the hands uncrossed and crossed over the body midline. Deafness appears to selectively affect the performance of the numerical task relying on verbal processes while keeping intact the task relying on visuospatial processes. Indeed, while a classic SNARC effect was found in all groups and in both hand postures of the number comparison task, deaf adults did not show the SNARC effect in both hand postures of the parity judgment task. These results are discussed in light of the spatial component characterizing the counting system used in sign language.

## Introduction

In humans, high level cognitive functions such as numerical cognition are considered to be grounded onto more basic sensorimotor functions^1^. A compelling demonstration of this idea resides in the association that occurs between numbers and space. In Western cultures, small numbers are indeed categorized faster with left-sided responses, while large numbers are categorized faster with right-sided responses^2^. This automatic association, known as the SNARC effect (i.e., Spatial Numerical Association of Response Codes), was observed in number comparison as well as in parity judgment tasks, and was first interpreted as an index of the visuospatial coding of magnitude^3,4^. Within this framework, the SNARC effect emerges because the preferential association between small-left and large-right is congruent with the left-to-right orientation of the mental number line^5–8^. Because the SNARC effect was also observed in a crossed hand posture (i.e., left/right hand in the right/left side of space)^5,9^, the coordinate frame in which the SNARC effect arises was assumed to be eye- or world-centred rather than hand-centred.

The mental number line framework has however been recently challenged by alternative hypotheses^10–12^. The first one assumes that the SNARC effect could emerge from verbal-spatial coding, namely, the mapping between the verbal associations of magnitude labels (small – large) and verbal-spatial labels (left–right). Based on the observation that the SNARC effect disappears under verbal load in parity judgment tasks and under spatial load in number comparison tasks^13,14^, the origin of the SNARC effect was assumed to depend on the task performed: verbal-spatial associations in parity judgment tasks; visuospatial coding in number comparison tasks. The second alternative hypothesis, named the “manumerical hypothesis”^15^, suggests that the association between numbers and space develops during childhood based on the direction of the finger counting habits.

Regardless the respective contribution of these alternative hypotheses, the study of deaf signers represents a unique opportunity to test the intrinsic relation between numbers and space, as sensorimotor interactions with the world around us are believed to influence the representation of numbers^16,17^. Deaf signers indeed use a visuo-manual language to communicate and therefore present a unique sensorimotor experience with the world. In contrast to oral language, sign language includes a highly significant visuospatial component (e.g., manual configurations to represent numbers^18^) and a phonology characterized by four visuospatial parameters of the signing hand (i.e., handshape, location, movement and orientation; see^19,20^). The primacy of visual cognition in deaf signers may therefore positively impact (or at least preserve) their visuospatial numerical skills^21^ (e.g., number comparison task). On the other hand, the fact that deaf individuals have less practice than their hearing peers in explicitly accessing the phonological representations of their native language^22^ (and often present a language delay) may negatively impact their verbal numerical abilities (e.g., parity judgment task).

Deaf individuals were accordingly reported to have poorer numerical abilities than their hearing peers (see^23^ for a review). They indeed show a delay of 2–3.5 years on mathematical achievement tests^24,25^. This delay is moreover more pronounced in tasks requiring verbal processes (for multiplicative reasoning, see^26,27^; for relational statements, see^28,29^; for fractions, see^30^). Despite their numerical difficulties, deaf individuals nevertheless show a classic SNARC effect^21,25,31^, with Arabic digits as well as with number signs^21^ (see^32^ for a systematic review). However, number comparison and parity judgment tasks were never compared in the same sample of participants. The spatial frame of reference that deaf use to map numbers onto space in different numerical tasks has therefore never been investigated so far.

In Belgium, deaf signers interestingly use a single hand (i.e., the dominant hand) to represent the numbers from 1 to 9. Number signs are therefore produced in one hemi-space, but are perceived in the other hemi space. As the stimulus–response compatibility effect can be determined by the effectors that are recruited for communication^21^ or finger-counting^15^ purposes (i.e., the hands), the particular way in which deaf individuals produce and perceive numbers on their fingers may thus lead to the use of a reference frame that differs from the one of hearing people. This difference between deaf and hearing individuals could moreover be specifically observed in verbal-spatial parity judgment tasks and be less salient in visuospatial comparison tasks.

To test these hypotheses, deaf signer, hearing signer and hearing control adults were asked to perform a visuospatial number comparison task as well as a verbal-spatial parity judgment task with the hands uncrossed and crossed over the body midline. If the spatial components of sign language have a positive impact on the visuospatial features of the mental number line, then deaf individuals should show a typical SNARC effect in the visuospatial number comparison task. In contrast, if typical verbal inputs are necessary for the development of the verbal-spatial features of numbers, then deafness should specifically affect the SNARC effect in the parity judgment task. By comparing the participants’ performances in the uncrossed and crossed hand’s postures, the frame of reference that deaf, hearing signers and hearing controls use to map numbers onto space will moreover be examined in both tasks. If the SNARC effect is observed even when participants cross their hand, it could be assumed that numbers are mapped onto an external frame of reference where small and large numbers facilitate responses in left and right side of space irrespective of the hand of response.

## Methods

### Participants

The study population consisted of twenty-one deaf individuals (12 women, 9 men, M_age_ = 39.0 ± 2.96 years), twenty hearing signers (15 women, 5 men; M_age_ = 37.8 ± 3.10 years) and twenty hearing controls (12 women, 8 men, 38.3 ± 3.29 years) (see Table 1 for a detailed description of the deaf participants). Hearing participants were Dutch or French native speakers, and deaf participants communicated in VGT (Vlaamse Gebarentaal: sign language used in the Dutch part of Belgium) or in LSFB (Langue des Signes de Belgique Francophone: sign language used in the French part of Belgium). None of the subjects reported neurological problems. Hearing controls and hearing signers were matched to deaf participants for age [*F(2, 58)* = *0.38, p* = *0.96, η*^2^ = *0.001*], gender, handedness, educational level [*F(2, 55)* = *1.91, p* = *0.16, η*^2^ = *0.065*] and mother tongue (French versus Dutch). Most deaf individuals reported sign language as their mother tongue. Six deaf participants reported acquiring sign language later in their life (7–20 years old), however they were fluent in sign language and indicated it as their preferred way of communication. Most of the hearing signers acquired sign language as a second (or multi) language with an average age of 18 years old for learning this language. Hearing signers had a minimum level of B1 for sign language. Both oral and written instructions in Dutch and in French were given. Additionally, instruction videos in sign language were presented to deaf participants. Written informed consents as well as information about the project and the rights of the participants were signed by all participants. The study was approved by the “Comité d’Ethique hospitalo-facultaire Saint-Luc-UCLouvain” (2019/19AOU/357) and the procedures were in line with the Declaration of Helsinki.

**Table 1:** Characteristics of deaf participants.

Subject	Age	Sex	Handedness	Onset		Cause
1	56	F	R	0		Hereditary
2	48	M	L	0		Rubella
3	23	F	R	0		Congenital
4	48	M	R	0		O_2_ insufficiency
5	51	F	R	0		Meningitis
6	28	M	R	0		Genetic
7	50	M	L	0		Genetic
8	37	F	R	0		Genetic
9	49	F	R	0		Rubella
10	23	F	R	0		Unknown
11	43	M	R	0		Hereditary
12	24	F	R	0		Unknown
13	20	M	R	0		Unknown
14	53	M	R	0		Hereditary
15	53	F	R	0		Hereditary
16	35	M	R	0		Unknown
17	63	F	R	0		Hereditary
18	35	M	L	0		Genetic
19	37	F	R	0		Nerf atrophy
20	22	F	R	0		Unknown
21	21	F	R	0		CMV

R, right-handed; L, left-handed; F, female; M, male; CMV, cytomegalovirus.

### Tasks and procedure

#### Number comparison task

In the number comparison task, participants had to decide whether a visually presented number was smaller or larger than 5. Only Arabic digits from 1 to 9 (except for 5) were used. The task comprised two conditions. In the first condition, the “smaller than 5” response was assigned to a left response button and the “larger than 5” response to a right response button (i.e., congruent condition). In the second condition, the reverse instruction was used: the “smaller than 5” response was assigned to the right response button and the “larger than 5” response to the left response button (i.e., incongruent condition). Participants had to execute the task with their hands in a parallel posture (i.e., uncrossed posture), and with the arms crossed over the body midline (i.e. crossed posture) [adapted from^33^].

#### Parity judgment task

In the parity judgment task, participants had to judge, with the hands uncrossed and crossed over the body midline, if a visually presented Arabic digit (from 1 to 9, except 5) was odd or even. In a first condition, the “odd” response was assigned to a left response button, and in a second condition the “odd” response was assigned to a right response button. In both conditions, half of the trials induced congruent responses (small numbers and a press of the left response button/large numbers and a press on the right response button) while the other half induced incongruent responses (small numbers and a press of the right response button/large numbers and a press of the left response button).

#### Procedure

The tasks required a response with the left or the right hand and was therefore bimanual. In both tasks, participants had to respond as quickly and as accurately as possible by pressing one of two response buttons (with their index, middle and ring fingers). The response buttons were placed 30 cm in front of their body and 20 cm away from the body midline in the left and right hemi-spaces. Each individual had to complete 4 blocks of trials (for both tasks) in which the order of response mode and posture conditions were counterbalanced across participants. Each visual numeral was randomly presented 10 times in each condition (total of 8 numerals × 10 presentations × 4 conditions = 320 stimuli) with an inter-stimuli interval from 1500 to 2500 ms. Stimuli were presented until the participants’ responses. Numbers were presented on a computer screen with size 88 and in font Courier New. Participants executed the task in a silent room and recording were taken using E-prime software 2.0 running on a Dell computer with Windows XP as operating system.

### Statistical analysis

Statistical analysis was executed using IBM SPSS statistics 26 software for Mac OS Monterey 12.0.1 (Armonk, NY). Statistical significance was set at *p* < 0.05 for all computations. Data were checked for normality of distribution by the Shapiro–Wilk test and presented as Mean ± Standard Error (SE). Accuracy scores and reaction times (ms) were measured.

Accuracy scores were presented as correct or not. A binary General Linear Mixed Model (GLMM) was then run on this measure with subjects as random factor and response button, hand posture and magnitude as random effects. Task (number comparison, parity judgment), group (deaf, hearing signers, hearing controls), hand posture (uncrossed, crossed), response button (left button, right button), and magnitude (small, large) were indicated as fixed main factors.

To normalize reaction times, a natural logarithmic transformation was applied on these values [ln(RT)]. The data were then analysed using a General Linear Mixed Model with ln(RT) as the dependent variable (only reactions times for correct answers were included); task, group, hand posture, response button, and magnitude as fixed factors. Subjects were indicated as random factor and response button, hand posture and magnitude were inserted as random effects. Similar GLMMs were performed afterwards per task due to a significant task × group × response button × hand posture × magnitude interaction [*F(65, 34.0)* = *2.46, p* = *0.000*]. Sequential Bonferroni adjusted significance level was adapted when appropriate. Outliers were removed from statistical analysis when 3 standard deviations out of the mean (calculated after logarithmic transformation). For the number comparison task, the proportion of outliers per group was: 0.98% in the deaf group, 0.97% in the hearing signers group, and 0.98% in the hearing controls group. For the parity judgment task, 0.98% of the data was removed for the deaf group, 0.99% for the hearing signers group, and 0.99% for the hearing controls group. From the total sample of participants, one deaf and two hearing controls were removed for the number comparison analysis due to missing results. Two deaf, one hearing signer and two hearing controls were removed from the parity judgment analysis due to missing results. For the GLMM concerning the two tasks, only the overlapping participants were taken into account.

## Results

The binary GLMM on the accuracy data did not converge since almost all answers were successful (*overall percent correct* = *98.6%*). No significant main effects were highlighted for task [*F(7, 35.0)* = *0.000, p* = *1.00*], response button [*F(1, 35.0)* = *0.001, p* = *0.98*], hand posture [*F(1, 35.0)* = *0.001, p* = *0.98*], magnitude [*F(1, 35.0)* = *0.006, p* = *1.00*], group [*F(2, 35.0)* = *0.000, p* = *1.00*], nor significant interactions.

The GLMM on the ln(RTs) indicated a main effect of response button [*F(1, 34.0)* = *42.42, p* = *0.000*], magnitude [*F(1, 34.0)* = *29.4, p* = *0.000*], hand posture [*F(1, 34.0)* = *48.7, p* = *0.000*]. No significant group difference was found [*F(2, 34.0)* = *0.65, p* = *0.52*], but several significant interactions were observed: task × group × response button × hand posture × magnitude [*F(65, 34.0)* = *2.42, p* = *0.000*], task × response button × magnitude [*F(7, 34.0)* = *15.5, p* = *0.000*], group × response button × magnitude [*F(2, 34.0)* = *4.75, p* = *0.009*], task × group [*F(14, 34.0)* = *13.9, p* = *0.000*], task × hand posture [*F(7, 34.0)* = *16.3, p* = *0.000*], task × magnitude [*F(7, 34.0)* = *3.17, p* = *0.002*], response button × hand posture [*F(1, 34.0)* = *14.2, p* = *0.000*], and response button × magnitude [*F(1, 34.0)* = *65.2, p* = *0.000*]. Because of the significant 5-way interaction, the authors decided to perform similar GLMMs on the ln(RT) data of each task separately.

### Number comparison task

Regarding the reaction times of the number comparison task, the GLMM showed a main effect of response button [*F(1, 18.3)* = *43.1, p* = *0.000*], magnitude [*F(1, 18.3)* = *24.9, p* = *0.000*], and hand posture [*F(1, 18.3)* = *45.7, p* = *0.000*]. No significant main effect for group was seen [*F(2, 18.3)* = *0.55, p* = *0.58*]. Two significant interactions were observed, namely a response button × hand posture interaction [*F(1, 18.3)* = *6.93, p* = *0.008*] and a response button × magnitude interaction [*F(1, 18.3)* = *54.1, p* = *0.000*]. The final GLMM was run with only the two significant interactions included and led to the same conclusion: Faster responses were, overall, observed with the right response button (*M* ± *SE* = *6.42* ± *0.017*) than the left response button (*M* ± *SE* = *6.47* ± *0.017*). The uncrossed hand posture showed faster reaction times (*M* ± *SE* = *6.41* ± *0.018*) than the crossed hand posture (*M* ± *SE* = *6.47* ± *0.018*). The small magnitude elicited faster responses (*M* ± *SE* = *6.43* ± *0.017)* than the large magnitudes (*M* ± *SE* = *6.46* ± *0.017*). Participants, overall, responded faster with the right button than the left button, but this right button advantage was bigger for larger numbers (*M* ± *SE* = *6.49* ± *0.018 for the left response button; M* ± *SE* = *6.42* ± *0.018 for the right response button; p* = *0.000*) than for smaller numbers (*M* ± *SE* = *6.44* ± *0.018 for the left response button; M* ± *SE* = *6.42* ± *0.018 for the right response button; p* = *0.005*). Furthermore, a more pronounced SNARC effect was observed in the uncrossed hand posture (*M* ± *SE* = *6.44* ± *0.018 for the left response button; M* ± *SE* = *6.38* ± *0.018 for the right response button; p* = *0.000*) compared to the crossed hand posture (*M* ± *SE* = *6.49* ± *0.018 for the left response button; M* ± *SE* = *6.54* ± *0.018 for the right response button; p* = *0.000*).

**Figure 1:**
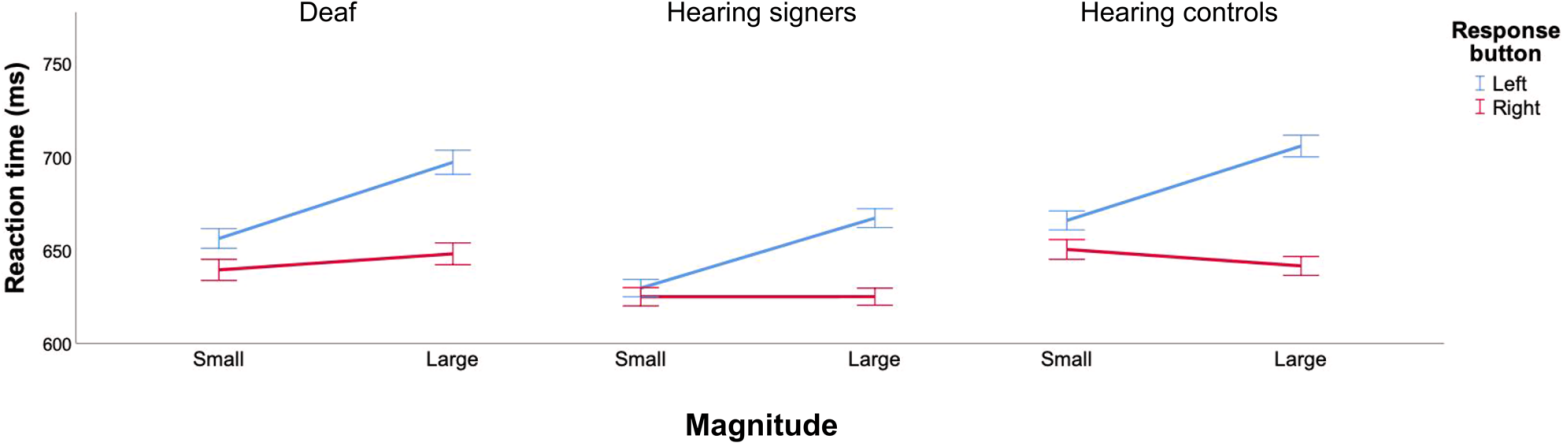
Mean reaction times for deaf (left graph), hearing signers (middle graph) and hearing controls (right graph) in the number comparison task. Error bars represent the standard error of the means. Untransformed reaction times are represented in the graph for the ease of comprehension.

Overall, all groups showed a classic SNARC effect when performing the number comparison task with uncrossed as well as with crossed hands (see Fig. 1), but this effect seems larger in the uncrossed position than in the crossed posture.

### Parity judgment task

The ln(RT) scores of the parity judgment task were also separately analysed using a GLMM. This last analysis showed a significant main effect of response button [*F(1, 17.7)* = *36.8, p* = *0.000*], hand posture [*F(1, 17.7)* = *24.9, p* = *0.000*], and magnitude [*F(1, 17.7)* = *9.69, p* = *0.002*]. Moreover, a significant group × response button × magnitude interaction [*F(2, 17.7)* = *4.55, p* = *0.011*], and a significant response button × magnitude interaction [*F(1, 17.7)* = *15.0, p* = *0.000*] were observed. No main group effect was observed [*F(2, 17.7)* = *1.06, p* = *0.35*]. The final GLMM was run including only the significant interaction and led to the same conclusion: In general, participants responded faster with the right response button (*M* ± *SE* = *6.50* ± *0.015*) than the left response button (*M* ± *SE* = *6.55* ± *0.015*). The uncrossed hand posture showed faster reaction times (*M* ± *SE* = *6.50* ± *0.015*) than the crossed hand posture (*M* ± *SE* = *6.55* ± *0.015*). The small magnitude elicited faster responses (*M* ± *SE* = *6.52* ± *0.015)* than the large magnitudes (*M* ± *SE* = *6.53* ± *0.015*). The right response button induced faster responses compared to the left response button, which were more pronounced for larger numbers (*M* ± *SE* = *6.56* ± *0.015 for the left response button; M* ± *SE* = *6.50* ± *0.015 for the right response button; p* = *0.000*) than for small numbers (*M* ± *SE* = *6.54* ± *0.015 for the left response button; M* ± *SE* = *6.50* ± *0.015 for the right response button; p* = *0.000*). The estimated means table comparing the two magnitudes across groups and across response buttons indicated a difference between the small and large magnitudes for the left response button in the hearing signers (*M* ± *SE* = *6.49* ± *0.027 for small magnitudes; M* ± *SE* = *6.53* ± *0.027 for large magnitudes; p* = *0.000*) and in the hearing controls group (*M* ± *SE* = *6.55* ± *0.027 for small magnitudes; M* ± *SE* = *6.59* ± *0.027 for large magnitudes; p* = *0.000*) group. No difference between the small and large magnitudes for the left or the right response button was seen in the deaf group (*For the left response button: M* ± *SE* = *6.56* ± *0.027 for small magnitudes; M* ± *SE* = *6.57* ± *0.027 for large magnitudes; p* = *0.29*).

**Figure 2:**
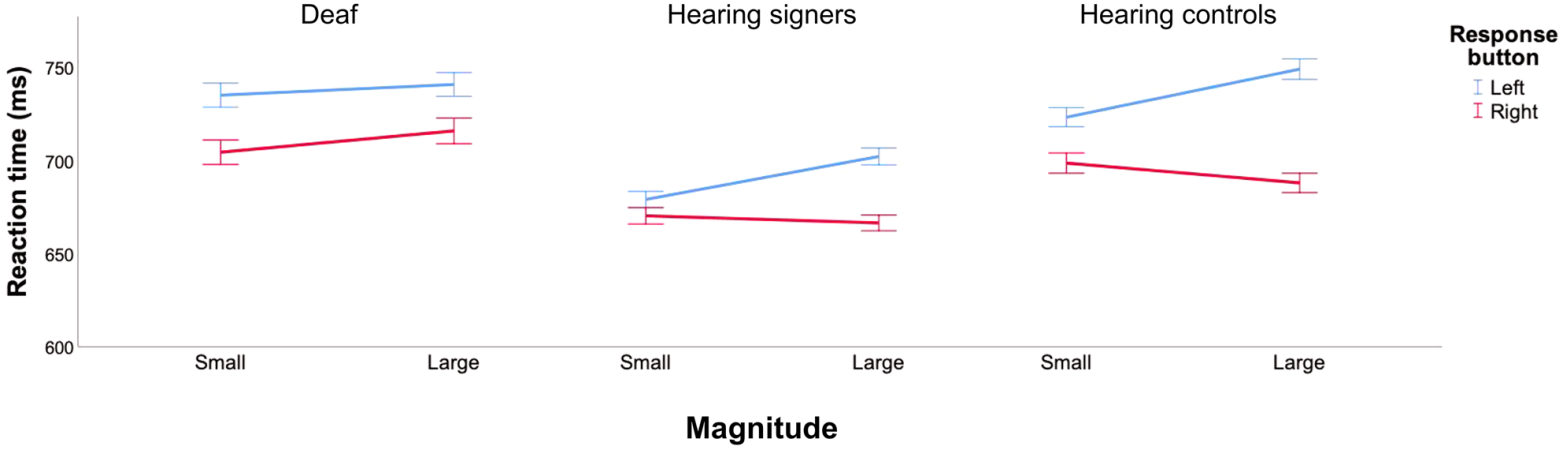
Mean reaction times for deaf (left graph), hearing signers (middle graph) and hearing controls (right graph) in the parity judgment task. Error bars represent the standard error of the means. Untransformed reaction times are represented in the graph for the ease of comprehension.

Hearing signers and hearing controls thus showed a classic SNARC effect when performing the parity judgment task in both hand postures. In contrast, when deaf participants performed the parity judgment task, the SNARC effect failed to appear (see Fig. 2).

## Discussion

The aim of this study was to further understand the development of number-space interactions in deaf signers who possess unique sensorimotor experiences. Deaf, hearing signer and hearing control adults were asked to perform a number comparison task and a parity judgment task with the hands uncrossed and crossed over the body midline. In this way, the emergence of a possible SNARC effect could have been registered. Several hypotheses have already been brought forward to explain this SNARC effect, as stated in the introduction. First, the SNARC effect could emerge due to the left-to-right orientation of the mental number line^5–8^. Second, verbal-spatial coding could influence the presence of a SNARC effect^10–14^. A manumerical hypothesis has finally been brought forward based on the finger counting habits developed as a child^15^. Our results are more in line with the verbal-spatial coding hypothesis.

In the number comparison task, deaf signers, hearing signers and hearing controls all showed a classic SNARC effect in the uncrossed and crossed hand posture conditions. All three groups showed faster responses for the right button, but this was especially true when larger numbers were presented. Our results therefore nicely support what was already reported by Bull and colleagues in 2005. In their study^2^, deaf participants also showed the SNARC effect when judging, with the hands uncrossed, whether Arabic digits from 1 to 9 were smaller or larger than 5. In 2006, the same researchers found the SNARC effect when ASL signers made magnitude decisions of size congruent Arabic and ASL numerals from 1 to 6^34^.

In the present study, a classic SNARC effect was observed even when participants crossed their hands. The SNARC effect was nevertheless less pronounced (but still present) in the crossed position. In the literature, this “crossing effect” has been interpreted as reflecting a conflict between the anatomical coordinates of the responding hand and the external coordinate of the stimulus that has to be processed. While the anatomical coordinate of the responding hand and the external coordinate of the response key were congruent in the uncrossed hands posture, they were incongruent in the crossed hands position (the left hand was placed in the right external space while the right hand was placed in the left external space)^9^. This misalignment has to be resolved before a response is executed and therefore decreases the strength of the interaction between numbers and space. We can therefore assume that numbers were mapped onto an external (eye- or world-centred) frame of reference in the three groups of participants. The usage of a visuospatial language by deaf individuals does therefore not change (or preserve) the nature of the external coordinate system in which numbers and space are mapped in number comparison tasks.

In the parity judgment task, a classic SNARC effect was observed in both hand postures in the two hearing groups of participants. However, and in contrast to what was observed in hearing signers and hearing controls, the deaf group did not show any SNARC effect in the crossed as well as in the uncrossed hand postures of the task. It has been previously suggested that retrieving the parity status of a number from the long-term memory results in a simultaneous retrieval of spatial associations^13^. In deaf, the linguistic counting system is presented in a manual counting format^35^. The particular way in which deaf individuals convey numbers on their fingers may thus lead to a verbal-spatial representation that differs from the one of hearing people. In Belgium more particularly, the signs to represent the numbers 1 to 9 in VGT and in LSFB are produced by using a single hand. This specificity of the Belgian sign languages may explain why we did not observe the SNARC effect in the parity judgment task. For deaf signers, the verbal associations of magnitude labels (small–large) and verbal-spatial labels (left–right) would not be so automatic as in hearing people. The use of one hand to sign numbers from 1 to 9 is however highly culture-specific. In German sign language, for example, the numbers larger than 5 are represented by two hands. Interestingly, a classic SNARC effect has already been reported in deaf signers performing a parity judgment task with German number signs from 1 to 9^31^. The discrepancy between the results of this study and our parity judgment data could therefore be explained by the specificities of the sign language used by the participants. Within this context, the mental representations of numbers in deaf individuals may correspond to the bodily spatial signing procedure^36–38^, meaning that deaf signers hold mental embodied representations of sign language signs (see also^39^). Another phenomenon to consider is that it is typical in signed face-to-face communication that the signer produces ordered sequences from left-to-right, while the observer perceives the signs with a right-to-left orientation. Competition between these two spatial orientations could possibly arise in the deaf population and therefore prevent the SNARC effect from clearly occurring in the parity judgment task.

In comparison to our study, research on blind participants showed classic SNARC effects when blind performed a parity judgment task in the uncrossed as well as in the crossed hand posture^33^. On the other hand, early blind individuals performing a number comparison task indicated a classic SNARC effect when the hands were uncrossed, but a reversed SNARC effect while crossing the hands over the body midline (see^32^ for a systematic review). The early blind thus showed a hand-based frame of reference (i.e., regardless of where in space the hands were placed, small numbers elicited faster left-hand responses while large numbers elicited faster right-hand responses), probably because early blindness prevents the use of an external coordinate system in space^33^. These results counteract nicely with the results of the current study and clearly indicate the role visual and verbal processes play in the development of number-space interactions.

## Conclusion

This study indicated a specific impact of deafness on number-space interactions. As hypothesized, deafness does not seem to influence the visuospatial features of the mental number line. A classic SNARC effect was indeed observed in both hand postures of the number comparison task. In contrast, deafness appears to selectively affect verbal-spatial task performance, as no SNARC effect was observed in both hand postures of the parity judgment task. These data further support the idea that different types of spatial information are engaged in different numerical tasks^13,14^ and show that specific sensorimotor experiences may shape the associations that occur between numbers and space.
